# Dynamics of bone healing after osteotomy with piezosurgery or conventional drilling – histomorphometrical, immunohistochemical, and molecular analysis

**DOI:** 10.1186/1479-5876-11-221

**Published:** 2013-09-23

**Authors:** Jônatas Caldeira Esteves, Elcio Marcantonio Jr, Ana Paula de Souza Faloni, Fernanda Regina Godoy Rocha, Rosemary Adriana Marcantonio, Katarzyna Wilk, Giuseppe Intini

**Affiliations:** 1 Department of Diagnostic and Sugery, Araraquara Dental School, Univ Estadual Paulista – UNESP, Araraquara, São Paulo, Brazil; 2 Department of Diagnosis and Surgery - Periodontics, UNESP - Univ Estadual Paulista, Araraquara Dental School, Araraquara, SP, Brazil; 3 Implantology Post Graduation Course, University Center of Araraquara–UNIARA, Araraquara, São Paulo, Brazil; 4 Department of Oral Medicine, Infection, and Immunity, Harvard School of Dental Medicine – Harvard University, 188 Longwood Avenue, Boston, MA 02115 – REB 403, USA; 5 Harvard Stem Cell Institute, Cambridge, MA, USA

**Keywords:** Piezosurgery, Bone healing, Osteotomy system, Bone surgery, Bone drilling

## Abstract

**Background:**

Piezosurgery is an osteotomy system used in medical and dental surgery. Many studies have proven clinical advantages of piezosurgery in terms of quality of cut, maneuverability, ease of use, and safety. However, few investigations have tested its superiority over the traditional osteotomy systems in terms of dynamics of bone healing. Therefore, the aim of this study was to evaluate the dynamics of bone healing after osteotomies with piezosurgery and to compare them with those associated to traditional bone drilling.

**Methods:**

One hundred and ten rats were divided into two groups with 55 animals each. The animals were anesthetized and the tibiae were surgically exposed to create defects 2 mm in diameter by using piezosurgery (Piezo group) and conventional drilling (Drill group). Animals were sacrificed at 3, 7, 14, 30 and 60 days post-surgery. Bone samples were collected and processed for histological, histomorphometrical, immunohistochemical, and molecular analysis. The histological analysis was performed at all time points (n = 8) whereas the histomorphometrical analysis was performed at 7, 14, 30 and 60 days post-surgery (n = 8). The immunolabeling was performed to detect Vascular Endothelial Growth Factor (VEGF), Caspase-3 (CAS-3), Osteoprotegerin (OPG), Receptor Activator of Nuclear Factor kappa-B Ligand (RANKL), and Osteocalcin (OC) at 3, 7, and 14 days (n = 3). For the molecular analysis, animals were sacrificed at 3, 7 and 14 days, total RNA was collected, and quantification of the expression of 21 genes related to BMP signaling, Wnt signaling, inflammation, osteogenenic and apoptotic pathways was performed by qRT-PCR (n = 5).

**Results:**

Histologically and histomorphometrically, bone healing was similar in both groups with the exception of a slightly higher amount of newly formed bone observed at 30 days after piezosurgery (p < 0.05). Immunohistochemical and qRT-PCR analyses didn’t detect significant differences in expression of all the proteins and most of the genes tested.

**Conclusions:**

Based on the results of our study we conclude that in a rat tibial bone defect model the bone healing dynamics after piezosurgery are comparable to those observed with conventional drilling.

## Abstract (pt)

**Resumo:**

**Contexto**

A piezocirurgia é um sistema de osteotomia utilizado em cirurgias médicas e odontológicas. Muitos estudos clínicos têm comprovado as vantagens da piezocirurgia em termos de qualidade de corte, manuseabilidade, facilidade de uso e segurança. Entretanto, poucos investigadores tem testado sua superioridade sobre os sistemas tradicionais de osteotomia em termos de dinâmicas de cicatrização óssea. Desta forma, o objetivo deste estudo foi avaliar as dinâmicas de reparo ósseo após osteotomias relizadas com cirurgia piezoelétrica e compará-las com a fresagem óssea tradicional.

**Métodos**

Cento e dez ratos foram divididos em dois grupos com 55 animais cada. Os animais foram anestesiados e as tíbias foram cirurgicamente expostas para criar um defeito de 2 milímetros de diâmetro utilizando cirurgia piezoelétrica (Grupo Piezo) e fresas convencionais (Grupo Fresa). Os animais foram sacrificados aos 3, 7, 14, 30 e 60 dias após a cirurgia. Amostras de osso foram coletadas e processadas para análises histomorfométrica, imunoistoquimica e molecular. A análise histomorfométrica foi executada em todos os períodos de avaliação (n = 8). Imunomarcação foi realizada para detecção de VEGF, CAS-3, OPG, RANKL and OC aos 3, 7 e 14 dias (n = 3). Para a análise molecular os animais foram sacrificados aos 3, 7 e 14 dias, RNA total foi coletado e procedeu-se à quantificação da expressão de 21 genes relacionados à via de sinalização BMP, via de sinalização Wnt, vias inflamatórias, osteogênicas e apoptóticas por meio de qRT-PCR (n = 5).

**Resultados**

Histológica e histomorfometricamente a cicatrização óssea foi similar em ambos os grupos à exceção de uma neoformação óssea discretamente maior observada aos 30 dias no grupo piezo (p < 0.05). As análises imunoistoquímica e por qRT-PCR não detectaram diferenças significantes entre todas as proteínas e genes testados.

**Conclusão**

Baseado nos resultados de nosso estudo nós concluímos que em um modelo de tíbia de rato a dinâmica de cicatrização óssea após piezocirurgia é comparável àquela observada com fresas convencionais.

## Background

Hard tissue cutting is a common procedure in the medical and dental fields, especially during orthopaedic, maxillofacial, and periodontal surgeries. Traditionally, rotating instruments such as burs have been used for osseous surgery. However, disadvantages are related to the use of these traditional systems, including bone overheating and damage to adjacent tissues [1,2]. Piezosurgery has been introduced as a valuable alternative to avoid disadvantages associated to the traditional rotating instruments.

Piezosurgery is performed by means of a device that uses microvibration at a frequency capable of cutting bone. Its mechanism of action is based on the ability of certain ceramics and crystals to deform when an electric current is passed across them, resulting in microvibration at ultrasonic frequency [3,4]. The vibration is then applied to a nitride-hardened or diamond-coated insert which moves at 25 – 30 KHz, a frequency that allows for selective cut of bone tissue [5]. Since its approval for commercial use in 2002, piezosurgery has been successfully utilized for many surgical procedures, such as maxillary sinus lifting [6], autologous bone graft harvesting [7], bone splitting [8], lateralization of the inferior alveolar nerve [9], and orthognathic and neurologic surgeries [5,10,11].

Clinical and pre-clinical studies combined with *in vitro* studies have shown that piezosurgery produces clean and precise osteotomies with smooth walls and decreased bleeding [12,13]. Maurer at al. [14] evaluated the micromorphological differences after using three osteotomy techniques and observed that different from rotatory drilling and saw, ultrasonic piezoelectric osteotomy preserved the original structure of the bone.

Few works however have studied the process of bone healing after piezosurgery and compared it to the bone healing that follows after osteotomy by traditional methods. A purely histological description was provided by Horton et al. [15]. These investigators described accelerated bone formation in alveolar defects generated by chisel and ultrasonic instrument in comparison to traditional drill. Later, Vercellotti et al. [16] evaluated the level of the alveolar bone crest after ostectomy with piezosurgery and burs in alveolar ridges of dogs. Histological analysis showed a bone level gain in the group treated with piezosurgery and bone loss in the diamond and carbide bur groups. A recent histomorphometrical study conducted by Ma et al. [17] compared the bone healing after osteotomies performed by piezosurgery versus osteotomies performed with oscillatory saws. They found no statistically significant differences in terms of histomorphometry. However, the authors found a higher degree of formation of vascularized tissues, of provisional matrix, and of bone remodeling activity at 7 and 14 days after use of piezoelectric surgery. The only *in vivo* study that combined histomorphometrical and molecular analysis was conducted by Preti et al. [18]. This group of investigators evaluated the level of osseointegration of titanium implants placed in surgical bed prepared with piezosurgery versus conventional drilling in tibiae of minipigs. They observed lower number of inflammatory cells, higher number of osteoblasts, increased expression of BMP-4 and TGF- β2, and lower expression of proinflammatory cytokines TNF-α, IL-1β and IL-10 in the piezosurgery group at 7 and 14 days after osteotomy.

Despite the extensive clinical use and proven efficacy of piezosurgery as an osteotomy system, the data presented in the literature to date does not provide a conclusive answer on whether piezosurgery presents with clear advantage over the traditional osteotomy systems with respect to bone healing acceleration. Data by Preti et al. [18] indicate that piezosurgery may accelerate the earlier phases of the implant osseointegration when compared to traditional drilling; however, a comprehensive study that evaluates and compares the bone healing process of a bone defect created with piezosurgery or other traditional systems is still missing. Thus, the aim of this study was to evaluate the dynamics of bone healing after piezosurgical and drilling osteotomy in bone defects. Our study hypothesized that bone healing after piezoelectric osteotomy is faster due to early enhanced expression of growth factors in comparison to conventional drilling. In order to test this hypothesis, the healing process of a subcritical bone defect was analyzed by histology and histomorphometry, immunohistochemistry (IHC), and genetic expression analysis of osteoblast differentiation regulators, osteogenic markers, inflammatory cytokines, and apoptotic factors. Our multifactorial analysis shows no significant differences in speed and quantity of bone regeneration when comparing piezosurgery over traditional drilling.

## Methods

### Animal studies

**Figure 1:**
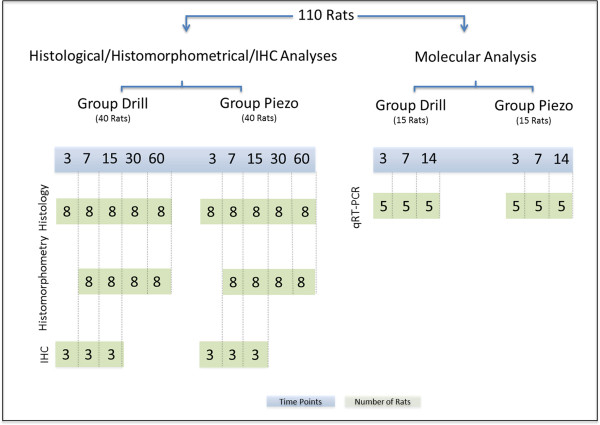
**Study design.** Different time points (days after surgery) are illustrated in blue and number of animals per group (n) are illustrated in green.

Ethical board approval was obtained for this study by the Ethics in Animal Research Committee of the School of Dentistry of Araraquara (UNESP, Brazil CEEA/FOAr 15/2010). One hundred and ten 3-month-old rats (*Rattus norvegicus albinus*, Holtzman) were used in this study. The rats were kept at a temperature of 22°C, in a 12 h light/dark cycle, with water and food ad libidum. After a 15-day acclimatization period, the animals were randomly assigned to the two experimental groups: Group I (Drill) and Group II (Piezo) with 55 rats each (Figure 1).

### Surgical procedure

All the animals were submitted to the same surgical procedure under general anesthesia with a combination of xylazine (0.04 ml/100 g body weight)(Francotar, Virbac do Brazil Ind. Com. Ltda., São Paulo, Brazil) and ketamine (0.08 ml/g body weight)(Vyrbaxil, Virbac do Brazil Ind. Com. Ltda., São Paulo, Brazil). Preoperative trichotomy of the inner region of the leg was performed and povidone iodine solution was applied to the surgical site to prevent possible sepsis. Next, an incision of approximately 20 mm in length was performed at the medial side of the right tibia, by the proximal metaphysis. Bone tissue was carefully exposed and a monocortical subcritical osteotomy of 2 mm in diameter was performed. For Group I, 2 mm in diameter drills were used to create the bone defect. For Group II a piezosurgery device – Piezo Master Surgery (EMS®, Nyon, Switzerland) - was set for cortical bone osteotomy (“surgical” mode, maximal cutting efficiency and 50% of sterile saline flow rate) and a 2 mm round diamond-coated tip was used to create the defect. External irrigation with sterile saline solution was provided in both groups. To facilitate the subsequent tissue processing, radio-opaque gutta-percha pins were positioned at a distance of 2 mm from the osteotomy edges. Soft tissue was sutured with 4–0 nylon (Ethicon, Division of Johnson & Johnson Medical Limited, São Jose dos Campos, São Paulo, Brazil). All the procedures were performed by the same surgeon, previously trained. After surgery, animals received an intramuscular dose of penicillin and streptomycin (0.1 ml/Kg of body weight) (Pentabiotic Pequeno Porte, Fort Dodges, Campinas, São Paulo, Brazil) and a gavage of acetaminophen (15 mg/Kg of body weight) (Paracetamol-Medley®, Campinas, Brazil).

### Collection of the samples

For histological, histomorphometric, and immunohistochemical (IHC) analysis tissues were collected at 3, 7, 14, 30 and 60 days post surgery (Figure 1, Histological/Histomorphometrical/IHC Analyses). More specifically, histology was performed on samples collected at 3, 7, 14, 30 and 60 days post surgery (n = 8) and histomorphometry was performed on samples collected at 7, 14, 30 and 60 days post surgery (n = 8). Immunohistochemical analysis was performed on tissues collected at 3, 7, and 14 days post surgery (n = 3). Block biopsies were harvested by collecting the treated area along with an additional 2 mm of surrounding tissue marked by the gutta-percha. Upon collection, tissue was fixed in 4% paraformaldehyde for 48 hours. The samples were then decalcified in EDTA buffered at pH 7.2 with 0.1 M sodium phosphate, embedded in paraffin, and cut into 4 μm sections along the longitudinal axes. For quantitative RT-PCR analysis, 5 animals per group were sacrificed at 3, 7 and 14 days after surgery (Figure 1, Molecular Analysis). Bone blocks were harvested by collecting the treated area along with the additional 2 mm of surrounding tissue marked by the guttaperca. The bone blocks were quickly rinsed once in PBS and immediately preserved in RNA Later (Sigma-Aldrich Inc. Brasil) until quantitative RT-PCR was performed.

### Histomorphometric analysis

The two most central histological sections of each bone defect were stained with hematoxylin and eosin for histomorphometric analysis. Standardized pictures were obtained with a digital camera (DSC295m, Leica Maicrosystems, Wetzlar Hessen, Germany) mounted on a microscope (Leica DM 2500, Leica Maicrosystems, Wetzlar Hessen, Germany). Images were analyzed using the Image J image analysis software [19] to quantify the bone neoformation. Analyses were repeated three different times at intervals of 1 week by the same blinded operator. Measurements were performed as follows: the total area (TA) to be analyzed was identified by delimiting the bone defect (2 mm in diamater) at 2 mm from the guttaperca reference points and the newly formed bone area (NFBA or bone neoformation) was then delineated within the TA. The percentage of NFBA was calculated according to the following formula: 100×NFBA(pixels)/TA(pixels) and values were submitted to statistic analysis using analysis of variance (ANOVA) followed by a post hoc Tukey’s test when the ANOVA suggested a significant difference among groups (*p* < 0.05).

### Immunohistochemical analysis

Immunohistochemical staining for Vascular Endothelial Growth Factor (VEGF), Caspase-3 (CAS-3), Osteoprotegerin (OPG), Receptor Activator of Nuclear Factor kappa-B Ligand (RANKL), and Osteocalcin (OC) was performed on 4-μm sections mounted on silanized slides (DAKO A/S, Golstrup, Denmark). Antigen retrieval for VEGF and CAS-3 detection was performed by incubation with 10 mM sodium citrate buffer, pH 6.0 at 70–75°C in a vapor cooker, for 30 min. Antigen retrival for OC, OPG and RANKL was performed by incubation with 0.5% trypsin for 20 minutes at 37°C. All sections were treated with 3% hydrogen peroxidase in methanol for 30 minutes to block endogenous peroxidase activity. Afterward, sections were incubated with 3% bovine serum albumin in phosphate buffered saline (PBS) (Sigma-Aldrich, St. Louis, MO) for 30 minutes at room temperature to block nonspecific protein binding. Subsequenlty, slides were incubated overnight with primary antibodies specific for CAS-3 (Rabbit polyclonal antibody – Abcam, Inc. USA, cat # ab44976, dilution 1:400), VEGF (Rabbit polyclonal antibody – Abcam, Inc. USA, cat # ab46154, dilution 1:400), OPG (Rabbit polyclonal antibody – Abcam, Inc. USA, cat # ab73400, dilution 1:300), RANKL (Mouse polyclonal antibody – Santa Cruz Biotechnology Inc., USA, cat # sc-7628, dilution 1:200), and OC (Mouse monoclonal antibody – Abcam, Inc. USA, cat # ab13420, dilution 1:200). For negative controls, the immunohistochemistry was performed by replacing the incubation step with primary antibodies with an incubation step with non-immune serum. Then, sections were incubated with biotinylated immunoglobulins (avidin-biotin complex, Universal LSAB 2 Kit/HRP kit, DAKO Inc., USA), and the reaction product was detected with an Avidin Biotin Peroxidase complex (ABC kit, DAKO Inc., USA) and stained with the chromogen substrate diaminobenzidine (Liquid DAB + Substrate Chromogen system, DAKO Inc., USA). Sections were counterstained with hematoxylin and examined by a calibrated examiner under light microscopy at x25 and x100 final magnifications. The quantification of the protein expression was performed by an ordinal qualitative analysis, following a previously published methodology [2,20]. Briefly, staining scores were categorized as follows: negative (−), positive (+), superpositive (++), and hyperpositive labels (+++). To perform a quantitative comparison, scores were then converted into percentile averages as follows: 0% (equivalent to “-“, negative staining), 20% (equivalent to “+”, 10% to 30% total staining), 60% (equivalent to “++”, 50% to 70% total staining), and 90% (equivalent to “+++”, 80% to 100% total staining). Percentile averages of each protein were submitted to statistical analysis using the non-parametric Mann–Whitney test, comparing the Drill group and the Piezo group at each time point. Significant difference between the groups was defined by p values < 0.05. Expression analyses of VEGF, RANKL, OPG, and OC were performed within the margins of the created defect. Expression analysis of Caspase-3 also included the scoring of bone areas surrounding the margins of the defect (2 mm).

### Quantitative RT-PCR analysis

Total RNA was extracted from bone samples using a Trizol reagent (Life Technologies Inc, USA) according to the manufacturer’s protocol. Complementary DNA was synthesized by reverse transcription of 1 μg of total RNA using oligo (dT) as primers (High Capacity cDNA synthesis kit, Applied Biosystems, Warrington, UK). Real-time quantitative PCR was conducted under standard enzyme and cycling conditions on a StepOne system (Life Technologies Inc, USA), using custom-designed real-time assays (Universal Probe Library - Roche, Indianapois, USA). According to the manufacturer’s instructions, reactions were performed in 10 μL triplicates for each target cDNA. Data was analyzed using a comparative ΔΔCt method [21]. Twentyone genes, divided into 5 categories were tested: 1) BMP Signaling: Bone Morphogenetic Protein 2 (*Bmp2* – NM_017178.1), Bone Morphogenetic Protein 4 (*Bmp4* – NM_012827.2), Runt related transcription factor 2 (*Runx2* – NM_053470.2), Noggin (*Noggin* NM_012990.1), Chordin (*Chordin* - NM_057134.1); 2) Wnt Signaling: Wingless-related MMTV integration site 5A (*Wnt5a* – NM_022631.1), Wingless-related MMTV integration site 10B (*Wnt10b* – NM_01108111.1), Lymphoid enhancer binding factor 1 (*Lef-1* – NM_NM_130429.1), Sclerostin (*Sclerostin* - NM_030584.1), Dickkopf Wnt signaling pathway inhibitor (*Dkk1* – NM_001106350.1); 3) Osteogenisis Markers: Collagen type 1 alpha (*Col1α* – NM_053304.1), Osteocalcin (*Oc* – NM_013414.1), Alkaline Phosphatase (*Alpl* – NM_013059.1), Osteoprotegerin (*Opg* – NM_U94330.1); 4) Inflammatory Cytokines and Apoptosis: Interleukin 1β (*IL-1β* – NM_031512.2), Interleukin 6 (*IL-6* – NM_012589.1), Tumor Necrosis Factor alpha (*Tnf-1α* – NM_012675.3), Caspase 3 (*Cas-3* – NM_012922.2); 5) Growth Factors: Platelet-derived growth factor (*Pdgf* – NM_031524.1), Transforming growth factor beta 1 (*Tgf-β1* – NM_021578.2) and Vascular endothelial growth factor (*Vegf* – NM_001110333.1). Gene expression levels were normalized to the housekeeping gene *β-actin* (NM_031144.2). At each time point (3 days, 7 days, and 14 days after surgery) values for each gene in the Piezo group were calculated as relative expression compared to the Drill group. Statistical analysis was performed using Student’s *t*-test and significant difference between the groups was defined by p values < 0.05.

## Results

### Histological and histomorphometrical analysis

**Figure 2:**
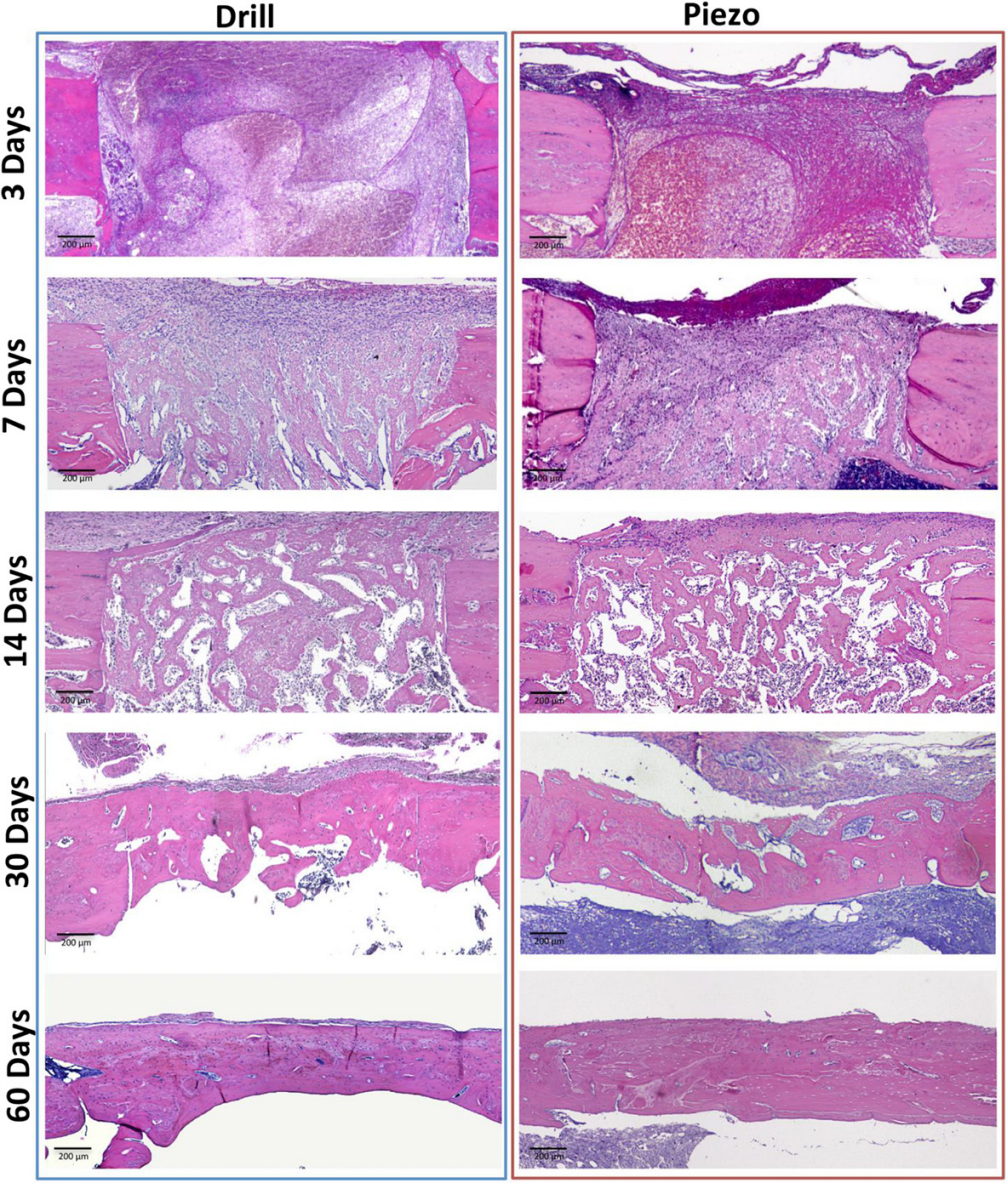
**Histological evaluation of the healing process over time.** Light micrographs obtained at 3, 7, 14, 30, and 60 days after surgery. Healing process after drilling (Drill group, left) and after piezosuregry (Piezo group, right). Hematoxylin and eosin staining.

**Figure 3:**
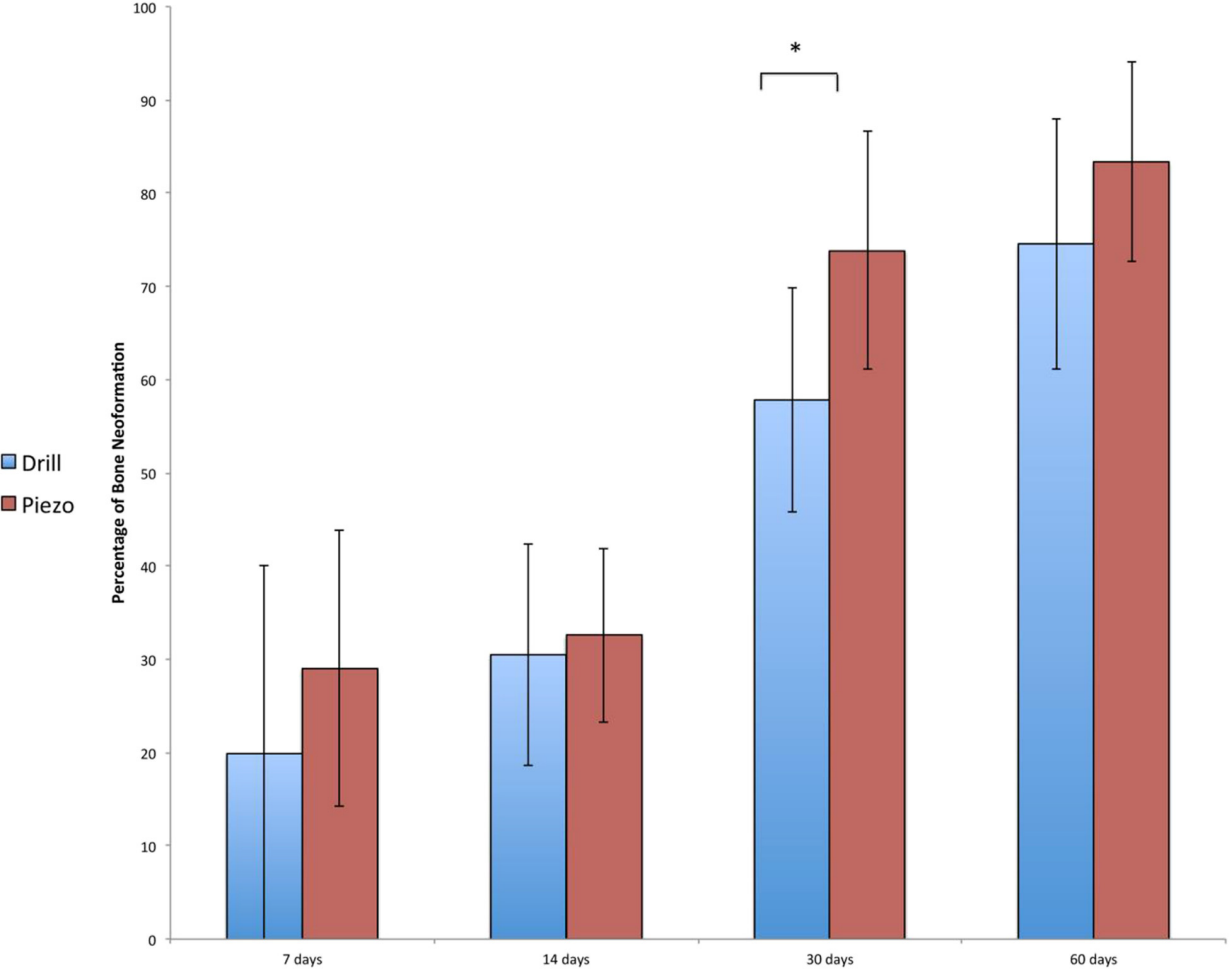
**Histomorphometrical analysis of the healing process over time.** Percentage of bone neoformation measured within the bone defects generated by drilling (Drill) or piezosuregry (Piezo). * indcates statistically significant difference between the Drill and Piezo group (p < 0.05, n = 8).

All subcritical bone defects in both groups healed with full regeneration of bone. The histological and histomorphometrical characteristics of the healing stages were very similar between the groups (Figures 2 and 3 respectively). Three days post surgery, the bone defects created by either drilling or piezosuregry showed regular shapes and well defined edges. In both cases, blood clot and inflammatory cells occupied the whole area of the defect and no bone neoformation was observed at this time. By day 7, the blood clot was progressively replaced by a connective tissue exhibiting a high cell density. Osteoid tissue was also present within the defect and bone neoformation was not statistically significant between the two groups at this time point. At 14 days post surgery the defects of both groups were mainly filled by newly formed woven bone with thin and irregular trabeculae surrounded by fibro-vascular tissue. At 30 days post surgery the piezosurgery osteotomies and drilling osteotomies were completely bridged by mineralized bone with irregular shape and volume. At this point, the amount of bone was significantly higher in the piezo group (*p* < 0.05, 73.88% ± 12.08 versus 57.81% ± 12.00). However, this difference disappeared at 60 days after surgery, when a mature strip of lamellar bone reconstituted completely the entire cortical thickness of the tibiae in both groups with no differences in terms of quantity and quality.

### Immunohistochemistry

**Figure 4:**
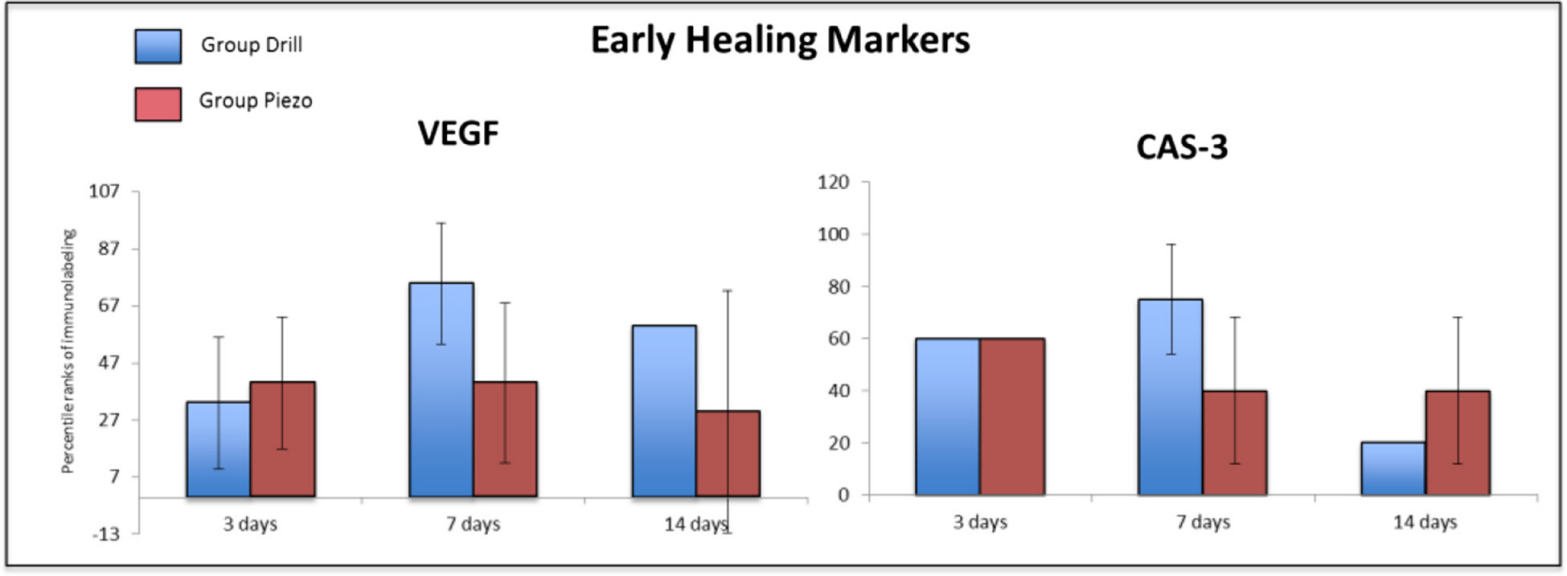
**Immonohistochemical analysis of early healing markers during the bone regeneration process.** Percentile ranks of immunolabeling for Vascular endothelial growth factor (VEGF) and Caspase-3 (CAS-3) at 3, 7, and 14 days after surgery, in bone defects generated by drilling (Drill) or piezosuregry (Piezo). No statistically significant differences were found across all time points (n = 3).

**Figure 5:**
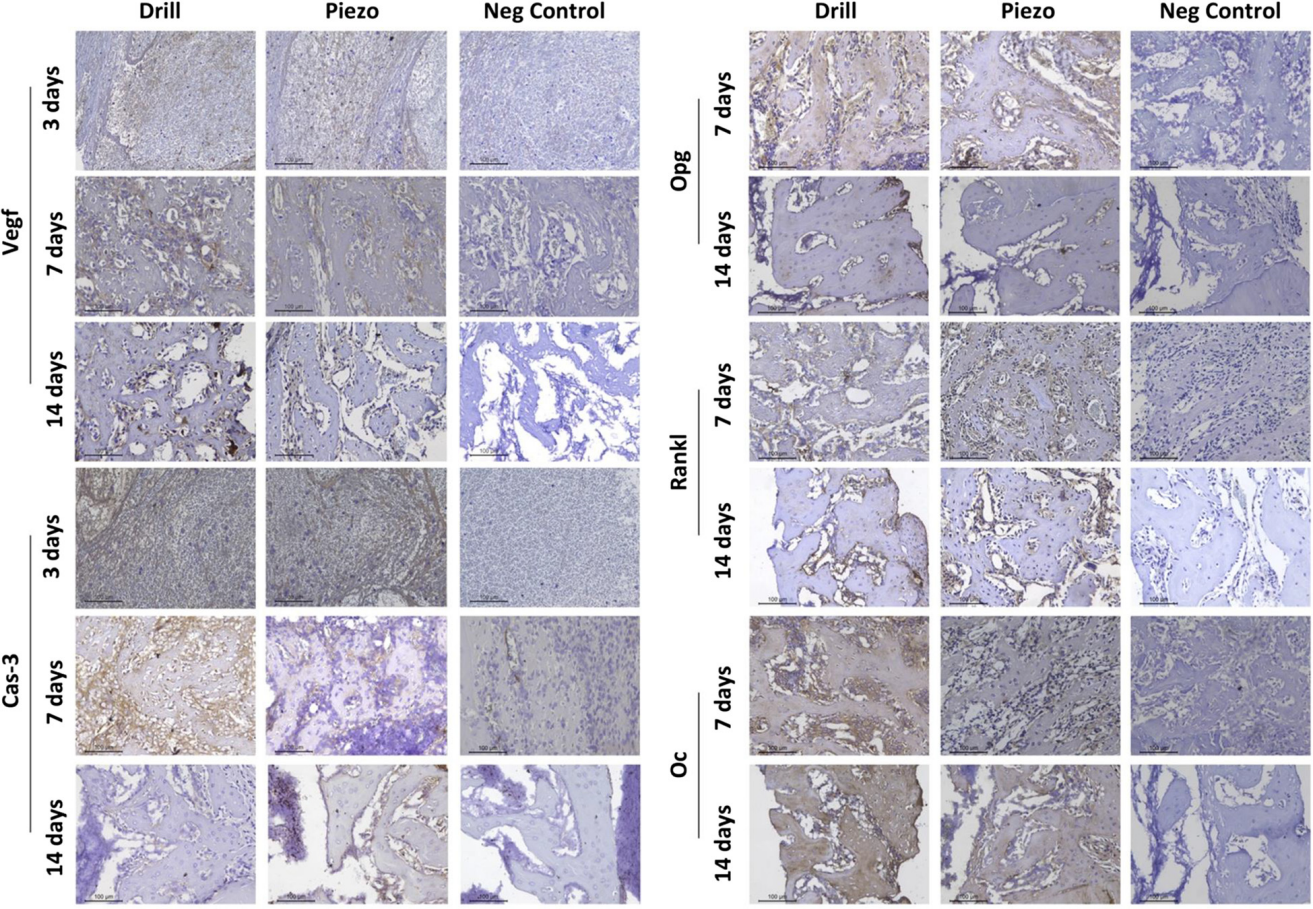
**Immunolabeling of early healing and bone remodeling markers.** Left: Immunolabeling of Vascular endothelial growth factor (VEGF) and Caspase-3 (CAS-3) of tissue sections obtained at 3, 7, and 14 days after drilling (Drill) or piezosuregry (Piezo). Right: Immunolabeling of Osteoprotegerin (OPG), Receptor activator of nuclear factor kappa-B ligand (RANKL), and Osteocalcin (OC) of tissue sections obtained at 7 and 14 days after drilling (Drill) or piezosuregry (Piezo). Sections were stained with the chromogen substrate diaminobenzidine and counterstained with hematoxylin. Staining scores were categorized as negative, positive (brown-yellow color), superpositive (brown color), and hyperpositive (intense brown color) (see Methods). Left (VEGF and CAS-3): at 3 days, only a few posivite regions in brown-yellow color are observed in both groups. Hyperpositive immunolabeling (intense brown color) is visible only at 7 days. At 14 days, the expression of VEGF and CAS-3 tended to be positive (brown-yellow) and superpositive (brown color). Right (OPG, RANKL and OC): at 7 days, hyperpositive immunostaining (intense brown color) is observed for OPG and OC, whereas superpositive immunoreaction (brown color) was detected for RANKL. At 14 days postsurgery, superpositive labeling (brown color) is observed for all three markers. In the negative controls no immunopositivity was detected at all times.

**Figure 6:**
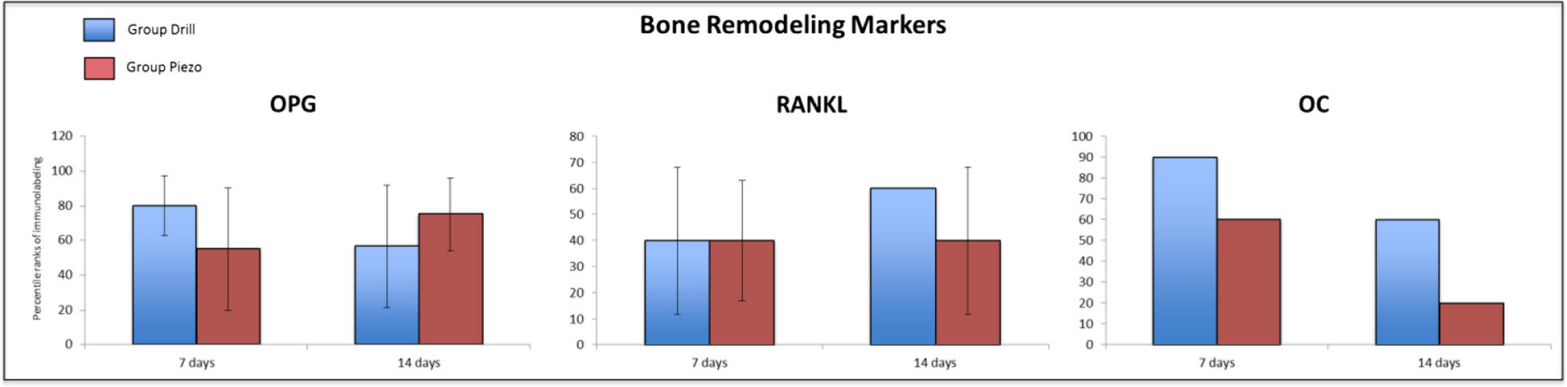
**Immonohistochemical analysis of bone remodeling markers during the bone regeneration process.** Percentile ranks of immunolabeling for Osteoprotegerin (OPG), Receptor activator of nuclear factor kappa-B ligand (RANKL), and Osteocalcin (OC) at 7 and 14 days after surgery, in bone defects generated by drilling (Drill) or piezosuregry (Piezo). No statistically significant differences were found across all time points (n = 3).

The expression of VEGF and CAS-3, two early stage markers of bone healing [22,23], was similar between the Drill and Piezo groups at 3, 7, and 14 days after surgery (Figures 4 and 5). The expression of OPG, RANKL and OC, three late stage markers of bone healing, was analyzed at 7 and 14 days after surgery (Figures 5 and 6). At both time points, no significant differences were detected for each gene between the two groups.

### Quantitative RT-PCR

**Figure 7:**
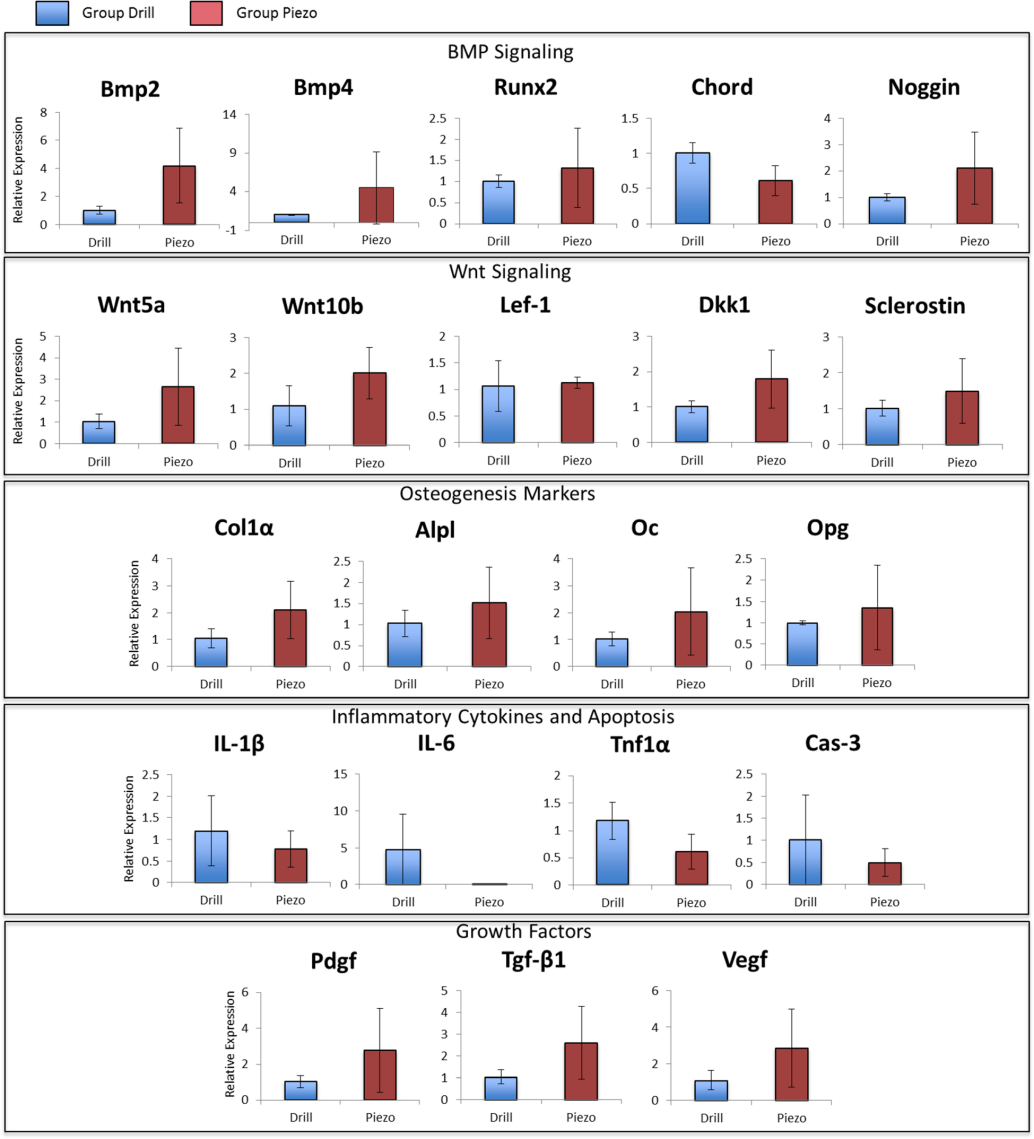
**Gene expression analysis at 3 days after surgery.** Comparative gene expression analysis between drilling (Drill) and piezosurgey (Piezo) of 21 genes involved with BMP signaling, Wnt signaling, inflammation, apoptosis and osteogenenis 3 days after surgery. * indicates statistically significant difference (p < 0.05), Nd (not detectable) indicates lack of detectable expression (n = 5).

**Figure 8:**
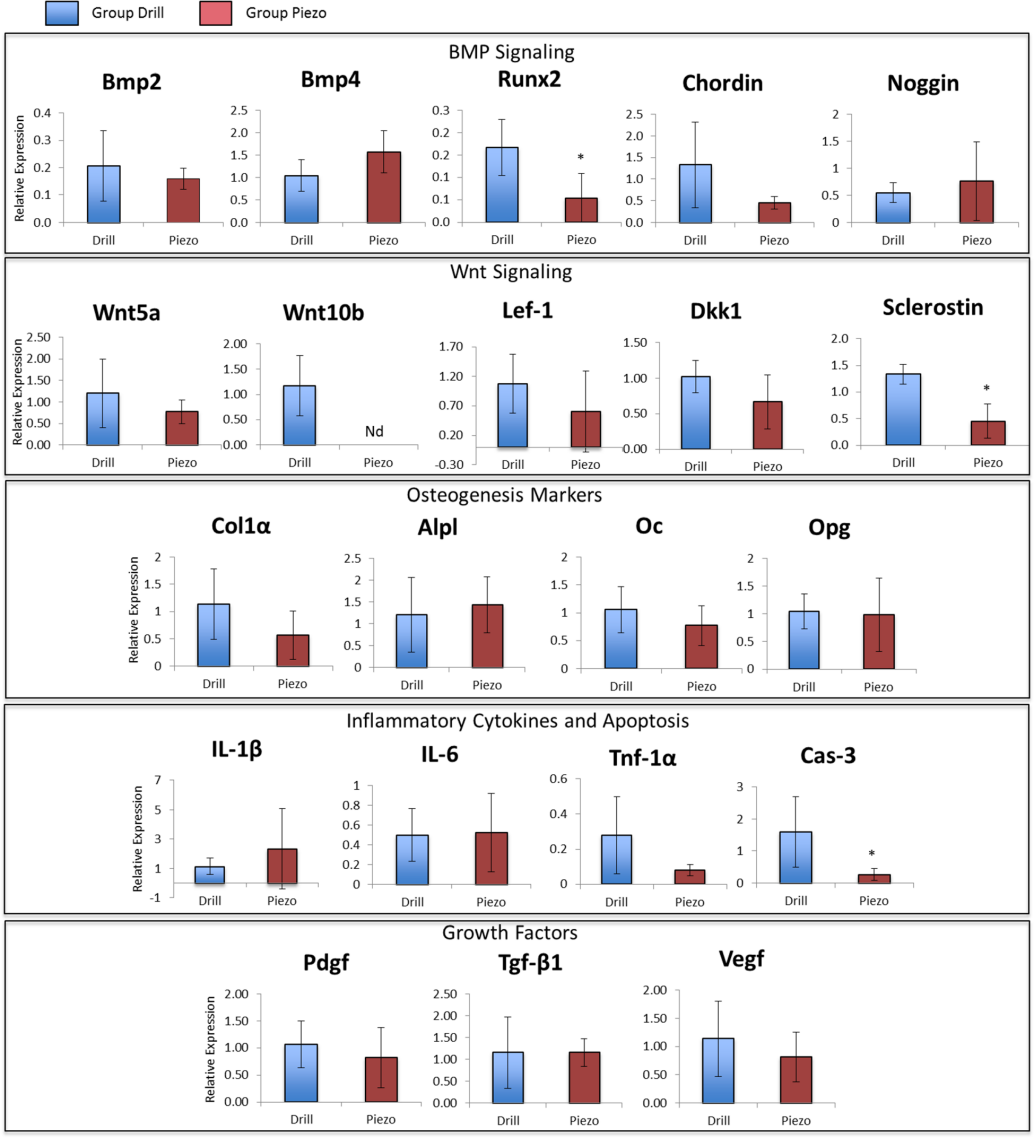
**Gene expression analysis at 7 days after surgery.** Comparative gene expression analysis between drilling (Drill) and piezosurgey (Piezo) of 21 genes involved with BMP signaling, Wnt signaling, inflammation, apoptosis and osteogenenis 7 days after surgery. * indicates statistically significant difference (p < 0.05), Nd (not detectable) indicates lack of detectable expression (n = 5).

**Figure 9:**
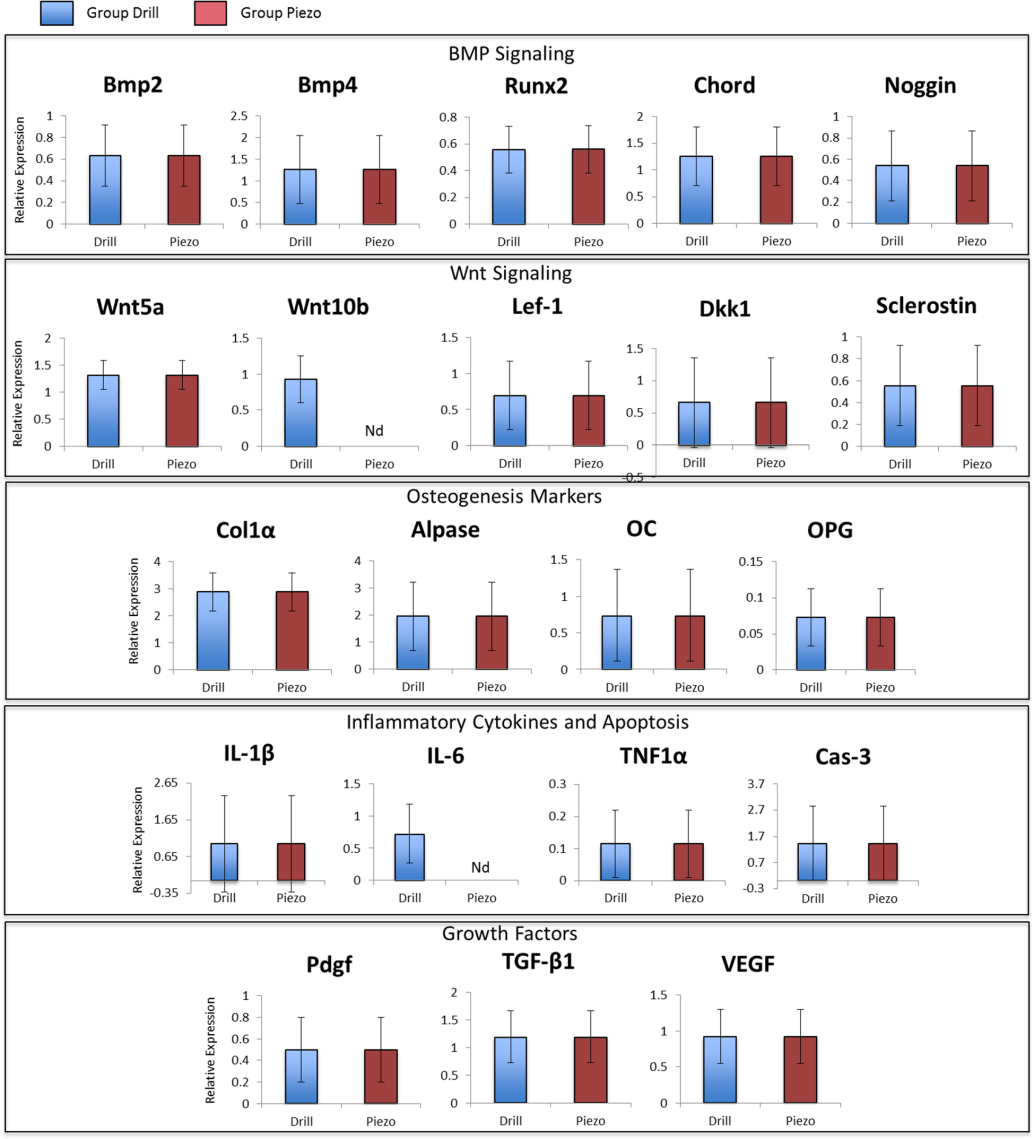
**Gene expression analysis at 14 days after surgery.** Comparative gene expression analysis between drilling (Drill) and piezosurgey (Piezo) of 21 genes involved with BMP signaling, Wnt signaling, inflammation, apoptosis and osteogenenis 14 days after surgery. * indicates statistically significant difference (p < 0.05), Nd (not detectable) indicates lack of detectable expression (n = 5).

Quantitative RT-PCR analysis was performed at 3, 7, and 14 days after surgery (Figures 7, 8, and 9 respectively) to evaluate expression of genes of the BMP and Wnt pathways and expression of genes that mark osteogenesis, inflammation, and apoptosis. Gene expression analysis of growth factors such *Pdgf*, *Tgf-β1*, and *Vegf*, which have also been shown to be involved with the bone healing process was also performed.

At three days after surgery (Figure 7), during the initial inflamamtory phase of healing when the bone defects are filled by inflammatory tissue (see Figure 2), the pattern of genetic expression was similar between the Drill and the Piezo group, with no statistically significant differences between the two groups for all the genes tested.

By 7 days (Figure 8), when bone formation is actively occurring and part of the defect is filled by newly regenerated bone (see Figure 2), *Runx2* (BMP signaling), *Wnt10b* and *Sclerostin* (Wnt signaling), and *Cas-3* (apoptosis) were differentially expressed, with no detectable levels of expression or statistically significant lower levels of expression in the Piezo group versus the Drill group (*p* < 0.05).

At 14 days (Figure 9), when the bone defects are filled with bone and highly vascularized fibro-fatty bone marrow (see Figure 2), *Wnt10b* (BMP signaling) and *IL-6* (inflammation) were differentially expressed, with no detectable levels of expression in the Piezo group.

## Discussion

The aim of this study was to evaluate the dynamics of bone healing after piezosurgical and drilling osteotomy. Our study hypothesized that when compared to conventional drilling, bone healing after piezosurgery is faster due to enhanced expression of proteins involved in bone regeneration and reduced expression of proteins involved in inflammation and apoptosis.

To test our hypothesis we chose to study the bone healing process of a subcritical bone osteotomy since subcritical bone defects spontaneously and consistently heal by complete regeneration of the missing bone as their bone regenerative potentials are always fully exploited [24]. The only variables that can influence their healing process are the level of inflammation that occurs immediately after the creation of the defect and the speed by which the regeneration process may occur. By means of this strategy, in a rat model of tibial subcritical size bone defect we therefore were able to follow and compare the events associated with the regeneration of bone defects created by drilling (Drill group) or piezosurgery (Piezo group).

We analyzed the bone formation by means of histology and histomorphometry at several time points, going from the early inflammatory stage (3 days after surgery) to the latest time point when complete bone regeneration and remodeling has occurred (60 days after surgery). By means of immunohistochemistry we also analyzed protein expression of early bone healing markers such as VEGF and CAS-3 at the early stages of the regenerative process (starting from day 3 up to day 14) and protein expression of bone remodeling markers such as OPG, RANKL, and OC at 7 and 14 days, when maximal regenerative activity occurs. Gene expression analysis of 21 genes expressing osteoblast differentiation regulators, osteogenic markers, inflammatory cytokines, and apoptotic factors was performed at 3, 7, and 14 days after surgery to validate and substantiate the immunohistochemical analysis. Among the osteoblast differentiation regulators, we tested several genes representative of the BMP canonical pathway [23] and of the Wnt canonical and non-canonical pathways [24] because of their relevance with the cellular activity that occurs during bone regeneration. Not all genes tested were consistently expressed throughout the healing process. We chose to utilize and show data regarding those genes that consistently presented with reproducible results.

The data collected showed that in our animal model the bone healing dynamics after piezosurgery are comparable to those observed with conventional drilling, with no evident signs of bone healing acceleration in the Piezo group versus the Drill group. At all the time points analyzed, histological analysis showed no differences between the defects created by piezosurgery and drilling. Histomorphometrical analysis also showed no differences’, with the exception of higher levels of newly regenerated bone at 30 days after piezosurgery. However, this difference disappeared at 60 days, when the amount of newly regenerated bone was equal for both groups. This result could be indicative of a better ability to regenerate bone of the Piezo group at 30 days of healing. However, our subsequent analyses indicate that this temporarily higher amount of bone formation is not due to healing acceleration during the early stages of healing. In fact, immunohistochemical analysis at 3 days after surgery showed no statistically significant difference in terms of expression of both VEGF and CAS-3 and gene expression analysis of 21 different genes, including *Vegf* and *Cas-3*, showed no significant differences for all the genes analyzed. Also, seven days after surgery, immunohistochemistry showed no differences in expression of VEGF, CAS-3, OPG, RANKL, and OC, indicating no changes during early healing in terms of vascularization, apoptosis, and bone regeneration and remodeling. The gene expression analysis at 7 days also showed no difference in expression of *Vegf*, *Cas-3*, *Opg*, and *Oc*. However, a significant reduction in expression of *Runx2*, *Wnt10b*, and *Sclerostin* was detected in the Piezo group at this time point. Since activation of BMP and Wnt signaling have been demonstrated to be essential at the early stages of bone repair [25,26], this data may indicate a reduction in terms of number of osteoprogenitor cells (reduction of *Runx2*) as well as a lower level of Wnt activity (lack of detection of *Wnt10b* indicating a direct down-regulation of the canonical Wnt pathway and reduction of expression of *Sclerostin* indicating the unnecessary expression of an inhibitor because of the already occurred down-regulation of the pathway) that may be interpreted as a deceleration rather than an acceleration of the healing process in the Piezo group. Furthermore, at 14 days after surgery, the immunoreaction also showed no significant differences between the two groups and gene expression analysis also showed no differences in expression of *Vegf*, *Cas-3*, *Opg*, and *Oc*. However, a significant reduction (lack of detection) of expression of *Wnt10b* and *IL-6* in the Piezo group was detected at this time point. Thus, data collected at each time point may be indicative of a deceleration rather than acceleration of the healing process associated to piezosurgery.

It could be speculated, however, that the decreased levels of expression of *Runx2* and *Wnt10b* seen at 7 and 14 days in the Piezo group are indicative of a diminished need for a full-speed regenerative process at these time points because in this group healing has already progressed to later and more advanced stages. However, considering that the histomorphometrical and immunohistochemical analyses did not show any difference in terms of amount and quality of bone regeneration at all early time points tested, we believe that this is not necessarily the case and that the gene expression changes observed in the Piezo group may simply represent normal variability of a complex process that is not regulated by few genes only.

Our data is not in contrast with the results shown by previous studies that compared piezosurgery with other traditional osteotomy methods. For instance, a study by Ma et al. [17] found no statistically significant differences in terms of histomorphometry but higher degree of formation of vascularized tissues, of provisional matrix, and of bone remodeling activity at 7 and 14 days after piezosurgery. These results may appear different from those shown in the present study. However, the animal model utilized by Ma and coworkers used bone defects smaller than those used in the present study and therefore the difference between the two studies may be due to the size of the bone defects utilized. Preti and co-workers [18] concluded that piezoelectric surgery appears to be more efficient in the first phases of bone healing than traditional osteotomy. Once again, these results may appear in contrast to those presented in this work. However, in their study Preti and co-workers analyzed the effects of piezosurgery on osseointegration of implants and not on regeneration of bigger bone defects. It is possible that the different conclusions are due to the different microenvironments studied. Similar consideration may be made between the bone defect microenvironment analyzed in our study and the periodontal defect microenvironment analyzed by Vercellotti et al. [16]. Thus, it remains possible that piezosurgery accelerates osseointegration of titanium implants and facilitates periodontal regeneration without being advantageous in terms of regeneration of bigger bone defects.

## Conclusions

Based on the results of our study we conclude that in a rat tibial subcritical bone defect model the bone healing dynamics after piezosurgery are comparable to those observed with conventional drilling. Further studies may be needed to analyze whether these two methods are comparable in terms of the healing dynamics of bone defects created in humans. However, piezosurgery remains a valuable alternative to the traditional rotating instruments thanks to its ease of use and bone cutting selectivity.

## Competing interests

All authors declare: no support from any comercial organization for the submitted work; no financial relationships with any organizations that might have an interest in the submitted work; no other relationships or activities that could appear to have influenced the submitted work.

## Authors’ contributions

JCE: Methods and experiments design, laboratory work, data collection and interpretation, manuscript writing; EMJr: Methods and experiments designs, results interpretation, supervision of the experimental work; APSF: Methods and experiments design (immunohistochemistry), laboratory experiments (immunohistochemistry), results interpretation, manuscript writing. FRGR: Methods design and samples processing (quantitative RT-PCR analysis), laboratory experiments (quantitative RT-PCR analysis). RAM: Methods and experiments designs, results interpretation, supervision of the experimental work; KW: Methods design and samples processing (quantitative RT-PCR analysis), laboratory experiments (quantitative RT-PCR analysis). GI: Conceptualization of the experiment, supervision of the experimental work, manuscript writing. All authors read and approved the final manuscript.
